# *Factor V Leiden, Prothrombin* and *MTHFR* Mutation in Patients with Preeclamsia, Intrauterine Growth Restriction and Placental Abruption

**DOI:** 10.3889/oamjms.2015.099

**Published:** 2015-09-18

**Authors:** Vesna Livrinova, Marija Hadzi Lega, Anita Hristova Dimcheva, Igor Samardziski, Rozalinda Isjanovska

**Affiliations:** 1 *University Clinic for Obstetrics and Gynecology, Faculty of Medicine, Ss. Cyril and Methodius University of Skopje, Skopje, Republic of Macedonia*; 2 *Institute for Transfusion Medicine, Faculty of Medicine, Ss. Cyril and Methodius University of Skopje, Skopje, Republic of Macedonia*; 3 *Institute for Epidemiology and Medical Biostatistics, Faculty of Medicine, Ss. Cyril and Methodius University of Skopje, Skopje, Republic of Macedonia*

**Keywords:** factor V Leiden, prothrombin, MTHFR, preeclampsia, IUGR, placental abruption

## Abstract

**BACKGROUND:**

*Factor V Leiden, Prothrombin* and *MTHFR* gene mutation, could have an influence in pregnancy with adverse outcome Preeclamsia, IUGR and Placental abruption.

**AIM:**

The aim of this study is to investigate the presence of above mentioned inherited thrombophilias and its statistical significance, distribution among the complicated and normal pregnancy, and relative risk for carrier of mutation to develop preeclampsia, IUGR and placental abruption.

**MATERIAL AND METHODS:**

Prospective cohort study is implemented at University Clinic for Obstetric and Gynecology in Skopje, Republic of Macedonia. The study included 109 delivered patients: 40 with preeclapmsia, 22 with IUGR, 17 with placental abruption and 30 as control group with normal pregnancy. The amount of 3 ml venous blood has been used for detection of these point mutations using ThromboStrip -Opegen, QIAGEN kit manufactured for thrombotic risk.

**RESULTS:**

The highest frequency was found: in the group with preeclampsia 35% were *MTHFR* homozygous, IUGR -*MTHFR* heterozygous 45%, Placental abruption- 52.9% *MTHFR* heterozygous, and in the control group without thrombophilia 56.7%. There were combined thrombophilia in 3 patients. There aren`t statistical significance in presence of thrombophilia among groups (p > 0.05). Statistical significance (p < 0.05) was found between carriers of *MTHFR* homozygous in preeclampsia and group with placental abruption and control group. Relative risk in IUGR group for *MTHFR* homozygous was 5.54 (1.37<RR<22.4). Relative risk in placental abruption for *Factor V Leiden* heterozygous was 4.50 (0.47<RR<42.75).

**CONCLUSION:**

The presence of mutation *MTHFR* homozygous could increase the risk for development of IUGR and mutation of *Factor V Leiden* for placental abruption. Further investigations with more patients are warranted.

## Introduction

Adequate fetomaternal circulating system is essential for normal development and function of placenta. It is obtained with mechanism which prevents coagulation of the maternal blood around chorionic villas and fetal blood in them [1]. Normal pathway in coagulation cascade includes balance between procoagulants, anticoagulant and fibrinolytic components in blood. Depend of the type of inherited thrombophilia, there is impaired neutralization of thrombin or failure to control generation of thrombin [2, 3]. This will cause malfunction of natural anticoagulants that maintain the fluidity of the blood. In normal circumstances, activated Factor V has procoagulant and anticoagulant activity in the same time. Activated Protein C inactivates factors Va and VIIIa and limiting the generation of thrombin. When gene for synthesis of factor V is mutated, there is *Arg506Gln* substitution, and one of the three cleavage sites for activated Protein C is inactive, without proteolysis inactivation of factor V.

On the other side factor V and factor VIII have augmentation effect for conversion of prothrombin to thrombin. Final effect is increased generation of thrombin and in vitro resistance to activated protein C to prolong activated partial-thromboplastine time [4, 5]. Mutation *G20210A* in 3` untranslated region of *Prothrombin* gene is associated with an increase level of plasma Prothrombin and consecutive excessive thrombin generation. In homozygous, hyperhomo-cisteinemia is as a result of *C677T* mutation in the gene for synthesis of MTHFR, lead to synthesis of thermo labile molecule of protein MTHFR with decrease enzyme activity in conversion of homocistein to metionin.

The pathogenesis for thrombophilia due to this mutation is still unknown [6]. The frequency of *FV Leiden* in white healthy individuals is 1%-15% in heterozygous and less than 1 % in homozygous [6, 7]. In Macedonia the frequency is 5.5% in general population, with difference between Macedonian population 6.9% and 2.9% in Albanian population, without statistical significant difference between the males and females [8]. Prothrombin gene mutation is 2.7%-7% and for *MTHFR* 5%-15% homozigous manner, and in 30-50% in heterozygous manner [9, 10]. These inherited thrombophilia substantially increased the risk for deep venous thrombosis and pulmonary thrombembolism during pregnancy and puerperium. Also they increase the risk for fetal loss after 20 weeks of gestation, especially after 28 weeks. In one more general study, it was found presence of 52% in pregnancy with preeclampsia, IUGR, placental abruption and stillbirth and they were heterozygous for *Factor V Leiden, prothrombin* gen mutation or homozygous for *MTHFR* gene mutation, as compared with 17% total of controls [11-13].

The aim of this study is to investigate the presence of above mentioned inherited thrombophilias and its statistical significance, distribution among the complicated and normal pregnancy, and relative risk for carrier of mutation to develop preeclampsia, IUGR and placental abruption.

## Material and Methods

This study was submitted and approved by the Ethical Review Committee of the Medical University in Skopje and is in adherence to the laws and regulations of the country in which the research was conducted. Written consent with patient permission was obtained from each patient.

This prospective cohort study was conducted at the University Clinic for Obstetric and Gynecology in Skopje, included 109 successively admitted and delivered patients during period of one year form March 2014 to March 2015. All delivered neonates were without sign of congenital infection, malformation and chromosomopathies.

The patients were distributed in four groups. The first group was consist from 40 patients with preeclamsia (PE), second group from 22 patients with intrauterine growth restriction (IUGR), third group from 17 patients with placental abruption (AP) and 30 patients as a control group of normal pregnancies and term spontaneous delivered healthy neonates. Inclusion criteria for PE was presence of proteinuria at least 0.5 g/L/24 hours, increase in systolic pressure for minimum 30 mmHg, and diastolic pressure 15 mmHg, measured two times apart for six hours, compared with blood pressure before pregnancy. Exclusion criteria were underlying pre existential morbidity: chronic hypertension, diabetes, renal disease, autoimmune and metabolic disease (NICE guidelines). Inclusion criteria for IUGR were birth weight less than 5^th^ percentile for gestational age and sex and exclusion criteria were presence of congenital infection, anomalies and chromosomopathies and mother who took medication, alcohol and with toxicomania. The placental abruption was clinically and/or histopatologicaly proven and exlusion criteria were rupture of membrane, uterine fibroid or other operation of uterus [13]. The differences between the numbers of participating patient are due to different frequency of each clinical entity. PE occurred in 8%-10%, IUGR 2%-3% and placental abruption 0.5%. During that period there were 5600 delivered patients in our clinic.

### Methods

**Figure 1:**
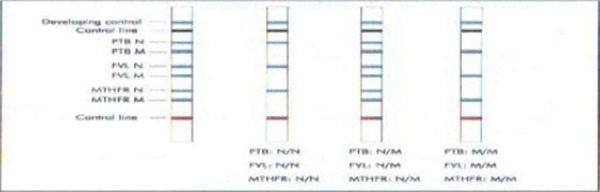
*Example of genotyping using ThromboStripqvality control*.

After delivery, 3 ml venous blood was taken from each patient with vacumtainer in epruvete with anticoagulant EDTA, and send to laboratory at Institute for Transfusion Medicine. For detection of mutations the laboratory used test: ThromboStrip-Opegen, from QIAGEN (molecular and immune diagnostic). This is a test for point gene mutations associated with venous thrombotic risk. ThromboStrip can detect three point mutations: *G1691A* for factor V, *G20210A* for *prothrombin* gene and *C677T* mutation for *MTHFR* gene. The procedure consists of these successive steps: DNA extraction, PCR amplification, hybridization, strip developing and detection. DNA extraction is manually from leucocytes from venous blood (spin protocol) with saline precipitation. After that, checking is preformed on 3% agarose gel for the presence fragments of free DNA. PCR was conducted on Ependorf (amplification of DNA fragments). Test membrane carried covalently attached DNA probes which specifically could recognize every gene amplificated sequence. There are two probe carriers for each gene –one normal and one mutated. The next phase is hybridization detection on machine AutoLipa 48, where probe carrier is specific attached for DNA fragments. The blue precipitation is shown at the place where hybridisation is. There are three possible results: no mutation, homozygous or heterozygous and it compares with control probe ThromboStrip on 3% agarous gel. Depends of appearance of blue band: one or two bands on test probe, the mutation are detected (Fig. 1). Sensitivity and specificity are limited only from the amount of DNA specimens (if there are 100 DNA fragments, compared with other methods, results concordant is 100%).

### Statistical method

SPSS V.20 was used for numeric and attributive paramatetars. Standard descriptive and analythical bivariant and multivariant methods were used. Statistical significans among attributive parameters was determined wit Chi-square test, and numerical parameters with Student’s t test.

## Results

A total of 109 patients were analyzed in this study. The patients were distributed in four groups - 40 patients with preeclampsia (PE), II- 22 patients with intrauterine growth restriction (IUGR), III- 17 patients with placental abruption (AP) and IV-30 patients as a control group of normal pregnancies and term spontaneous delivered healthy neonates.

Demographic data are presented: patient age-years (see Table 1) and ethnicity (see Table 2).

**Table 1:** Age distribution

Group	Mean	No	SD.	Minium	Maximum
**I**	29.4	40	6.9	18.0	43.0

**II**	31.2	22	5.8	21.0	42.0

**III**	31.5	17	5.7	20.0	42.0

**IV**	30.1	30	4.2	22.0	41.0

**Total**	30.3	109	5.8	18.0	43.0

**Table 2:** Ethnical distribution

Ethnicity/group	I		II		III		KG-IV	

	N	%	N	%	N	%	N	%
Macedonians	18	45.0	7	31.8	7	41.2	19	63.3

Albanians	18	45.0	14	63.7	8	47.0	6	20.0

Gypsy	3	7.5	0	0	0	0	2	6.7

Bosnians	1	2.5	1	4.5	1	5.9	3	10.0

Turkish	0	0	0	0	1	5.9	0	0

Total	40	100.0	22	100.0	17	100.0	30	100.0

The differences between mean values in patient age in four groups aren’t statistical significant (F=0.730792, p=0.54).

The percent of Albanian and Gipsy population in the group with PE is above of their presence in national structure of population in Macedonia. In control group - 63.3% is Macedonian, Albanians - 20.0%, Gipsy - 6.7% and Bosnians - 10.0%. This distribution is similar with national structure of population in Macedonia.

**Figure 2:**
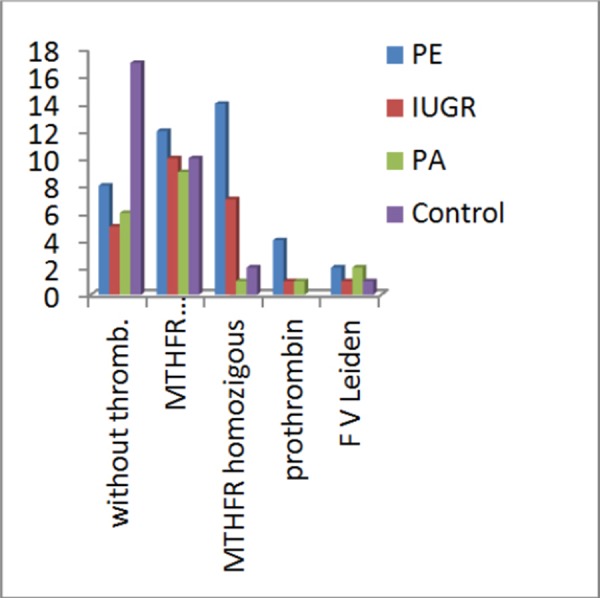
*Distribution of patient’s combination of clinical entities and presence of thrombophilia*.

Distribution of patient’s combination of clinical entities and presence of thrombophilia are presented in Table 3 and Figure 2.

**Table 3:** Distribution of patient’s combination of clinical entities and presence of thrombophilia

Thrombophilia type	I=40		II==22		III=17		IV=30	

	N	%	N	%	N	%	N	%
No	8	20.0	5	22.7	6	35.3	17	56.7

*MTHFR* heterozigous	12	30.0	10	45.5	9	52.9	10	33.3

*MTHFR* homozigous	14	35.0	7	31.8	1	5.9	2	6.7

*Prothrombin* heterozigous	4	10.0	1	4.5	1	5.9	0	0

*Factor V Leiden* heterozigous	2	5.0	1	4.5	2	11.8	1	3.3

In the group with preeclampsia 35% of patient were *MTHFR* homozygous, 30% *MTHFR* heterozygous, *Prothrombin* heterozygous 10%, *FV Leiden* 5% heterozygous and without thrombophilia 20%.

In the group with IUGR, 31.8% of patient were *MTHFR* homozygous, 45.5% *MTHFR* heterozygous, *Prothrombin* heterozygous and *FV Leiden* heterozygous 4.5% (coinheritance) and without thrombophilia 22.7%.

In the group with placental abruption, 5.9% of patient were *MTHFR* homozygous and *prothrombin* heterozigous (coinheritance), 52.9% *MTHFR* heterozygous, *FV Leiden* heterozygous 11.8% and without thrombophilia 35.3%.

In control group (healthy individuals) 6.7% of patient were *MTHFR* homozygous, 33.3% *MTHFR* heterozygous, *FV Leiden* 3.3% heterozygous and without thrombophilia 56.7%. Differences in frequencies present thrombophilia among four groups are without statistical significance for p >0.05.

There was coinheritance in two patients in group with IUGR: one with *MTHFR* homozygous, *Prothrombin* heterozygous and *FV Leiden* heterozygous. Another patient from the same group has coinheritance of *MTHFR* and protrombin heterozygous.

The third patient with coinheritance - *MTHFR* and *FV Leiden* heterozygous was in the group with placental abruption.

Statistical significance for p<0.05 was found in *MTHFR* homozygous between group with preeclampsia and placental abruption and control group. Statistical significance for p<0.05 was found in absence of thrombophilia between control group and the group with placental abruption and preeclampsia.

RR in PE group for *MTHFR* heterozygous, *MTHFR* homozygous and *FV Leiden* heterozygous was 1.7, 2.73 and 3.06 respectively. RR in IUGR group for *MTHFR* heterozygous, *MTHFR* homozygous and F V Leiden heterozigous was 1.8, 5.54 and 2.2 respectively. RR in group with placental abruption for *MTHFR* heterozygous, *MTHFR* homozygous and *FV Leiden* heterozygous was 1.62, 1.36 and 4.5 respectively.

**Table 4:** Multiple regression analysis

Independent variables	r = 0.274, r^2^ = 0.075 F = 2.841, p = 0.041		

	Beta	t – test	p - level
Age	-0.062399	-0.638054	0.525

Poor obstetric background	-0.040180	-0.416275	0.678

Positive familial anamnesis	0.272288	2.862150	0.005

*statistical significant (importance).

Statistical insignificancy between ethnical origin and type of thrombophilia was found in the group with preeclampsia and IUGR. In the third group with placental abruption, depends exists of ethnic origin and thrombophilia, with p<0.05, in Albanian population.

The differences among thrombophilia in the means value for age of patients is without statistical signifignance (P>0.05)

With multiple regression analysis it was concluded correlation between thrombophilia (criteria depend variable) and system of predictor’s variables of interest: age, familiar anamnesis and obstetrical history (independent variables), coefficient of multiple correlations (r) is 0.274. Coefficient of determination (r^2^) is 0.075, shown that all independent variables together have an influence in variability of thrombophilia with 7.5%, unless 92.5% belong to influence of other factors. Importance of multiple correlation coefficient tested on the base of F -distribution, shown the fact that influence of the predicators system of variables on thrombophilia (depend variable), is statisticaly significant for p = 0.041.

With analysis of each variables, it was concluded that important role has positive familiar anamnesis for p = 0.005.

## Discussion

The impact of inherited thrombophilia in pregnancy is investigated from many authors. Broad spectre of results could be found in literature. Review articles clearly shown the reasons for that finding. The most of them include patients with eclampsia, HELLP syndrome, severe PE, IUGR and placental abruption who were delivered in tertiary care hospitals.

Comparing the results from this study, it could be concluded that in healthy individuals the most frequent mutation is for *MTHFR* heterozigous, which is similar with the studies from other authors [17] but without thrombophilia were 56.7%, compared with the study of Kumferminc -80%. It was found no statistical significant difference between ethnical origin and thrombophilia in population in Macedonia [8].

*Factor V Leiden* mutation increased the risk for Preeclampsia has RR form 2.2-6.1 [6, 13-15] compared with RR-3.06 in this study. The most of the studies included patients with PE before 34 gw [16].

The relative risk for carriers of *Factor V Leiden* mutation was 4.5 in the group with placental abruption, but without statistical significance for other mutations.

Statistical significance for p<0.05 was found in *MTHFR* homozygous between group with preeclampsia and placental abruption and control group.

Statistical significance for p<0.05 was found in absence of thrombophilia between control group and the group with placental abruption and preeclampsia. The relative risks for IUGR for mutation: *FV Leiden* and *Prothrombin* are 2.58 and 2.03, p>0.05 [18, 22].

Only consistent result was found between carrier of *FV Leiden* homozygous and combined gene mutations and deep venous thrombosis in pregnancy and puerperium, where the risk increase to 40 folds [19-21].

In patients with placental abruption, was found RR of 3.9-5.0 for *Factor V Leiden* mutation [12, 22, 24].

These differences in the results probably are due to different inclusion criteria, the number of patients and selection bias and also because of ethnical background of some thrombophilia [23].

In conclusion, thrombophilia still remain field for further investigations, because a lot of studies shown that clinical expression in patients with thrombophilia are an interrelation between gene-age-environmental circumstances. It is important because the doctor should be offered screening for the patients with risks to develop complications in pregnancy [25].
